# Poly(styrene-co-butadiene)/Maghnia-Organo-Montmorillonite Clay Nanocomposite. Preparation, Properties and Application as Membrane in the Separation of Methanol/Toluene Azeotropic Mixture by Pervaporation

**DOI:** 10.3390/membranes11120921

**Published:** 2021-11-24

**Authors:** Amina Allel, Hassiba Benguergoura, Mohamed Wahib Naceur, Alain Ledoux, Waseem Sharaf Saeed, Taïeb Aouak

**Affiliations:** 1 Laboratoire Eau, Environnement, et Développement Durable (2E2D), Département de Génie des Procédés, Faculté de Technologies, Université Saâd Dahlab Blida 1, Route de Soumâa, B.P. 270, Blida 09000, Algeria; amina.allel@univ-blida.dz (A.A.); wnaceur@hotmail.com (M.W.N.); 2 Laboratoire de Chimie-Physique Moléculaire et Macromoléculaire LCPMM, Département de Chimie, Faculté des Sciences, Université Saâd Dahlab Blida 1, Route de Soumâa, B.P. 270, Blida 09000, Algeria; h_benguergoura@hotmail.com; 3 INSA de Rouen, LSPC, Normandie Université, 76801 Saint Etienne du Rouvray, France; alain.ledoux@insa-rouen.fr; 4 Chemistry Department, College of Science, King Saud University, P.O. Box 2455, Riyadh 11451, Saudi Arabia

**Keywords:** poly(styrene-co-butadiene), pervaporation, Maghnia-organo-montmorillonite clay, nanocomposite

## Abstract

In order to improve the thermal and mechanical properties of poly(styrene-co-butadiene) (SBR) to use it as a pervaporation membrane in the separation of the azeotropic mixture toluene/methanol, poly(styrene-co-butadiene) crosslinked Maghnia-organo-montmonrillonite (CSBR/OMMT), a nanocomposite of different compositions was first prepared by a solvent casting method. SBR was crosslinked in situ in the presence of OMMT nanoparticles by an efficient vulcanization technique using sulfur as a crosslinking agent and zinc diethyldithiocarbamate as a catalyst. The structure and morphology of the hybrid materials obtained were characterized by Fourier transform infrared spectroscopy, X-ray diffraction, and scanning electron microscope analysis. The thermal properties of these hybrid materials were studied by differential scanning calorimetry and thermogravimetric analysis/thermal differential analysis. The mechanical properties were studied by strength measurements. The results obtained occurred when the OMMT was incorporated in the CSBR matrix; a significant increase in the glass transition temperature of the SBR was observed which passed from −27 °C for virgin SBR to −21.5 °C for that containing 12 wt% of OMMT. The addition of OMMT nanoparticles to CSBR also improved the mechanical properties of this copolymer. When the OMMT content in the CSBR varied from 0 to 15% by weight, the tensile strength, the elongation at the nose and the modulus at 100% elongation increased from 3.45 to 6.25 MPa, from 162, 17 to 347.20% and 1.75 to 3.0 MPa, respectively. The results of pervaporation revealed that when the OMMT content varied between 3% and 12%, a significant increase in the total flux, the separation factor and the separation index by pervaporation increased from 260.67 to g m^−2^ h^−1^, 0.31 to 1.43, and 0.47 to 113.81 g m^−2^ h^−1^, respectively.

## 1. Introduction

Styrene–butadiene rubber (SBR) is mainly synthesized by free radical copolymerization of styrene with butadiene [1,2]. This copolymer is widely used in tire manufacturing due to its good resistance to cracking and abrasion. This material, compared to natural and synthetic rubbers, has desirable properties, such as insulation, air tightness, oil resistance, and resistance to low and high temperatures [3,4,5].

**Scheme 1:**
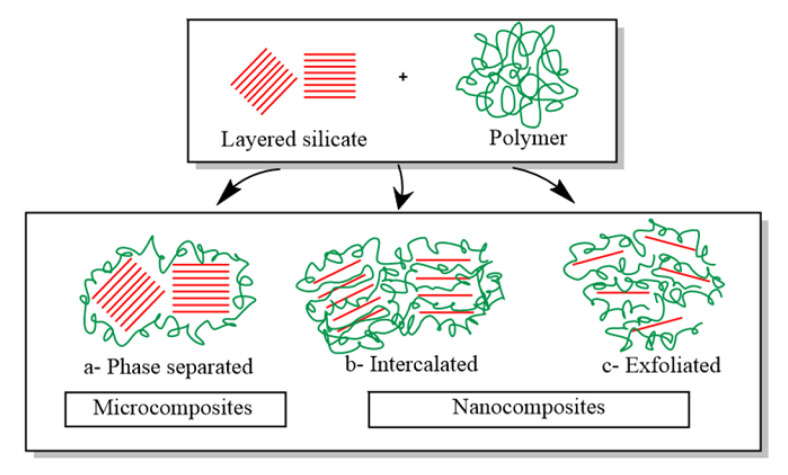
Types of polymer/clay composite structures: (**a**) microcomposites; (**b**) intercalated nanocomposites; (**c**) exfoliated nanocomposites.

Bentonite, which belongs to the family of clays, is known as a better reinforcing agent used in rubber compounds. Its low cost, low weight, and environmentally friendly reinforcing filler is still a challenge in the rubber industry [6]. It also improves the mechanical strength and thermal properties of SBR [7,8,9,10]. Bentonite clay has already improved the organic selectivity of glassy polymers [11]. When bentonite is incorporated into the polymer matrix, three types of materials can be generated depending on the how the clay particles are dispersed in the polymer matrix as shown in Scheme 1 [12]. 

Conventional composites are obtained when the polymer is unable to intercalate (or penetrate) between the silicate sheets, and a phase-separated composite is obtained (Scheme 1a); the intercalated nanocomposites, in which there are regular insertions of the polymer between the clay layers (Scheme 1b); and the delaminated (or exfoliated) nanocomposites, in which thin layers are dispersed in a polymer matrix (Scheme 1c) [13].

The latter structure is the most interesting because of the greater clay–polymer interactions, which produce composites with much better mechanical, thermal and barrier properties [14]. 

Nanocomposites involving SBR and clay are among the most interesting materials for researchers, especially during the last decade. Indeed, based on the character of separating clay into monolayers with a thickness of 1 nm, Zhang et al. [15] prepared SBR/clay nanocomposites by mixing SBR latex with clay/water dispersion.

The prepared hybrid materials showed that the main structure of the clay dispersion in the SBR was a bundle of layers, the thickness of which was 4–10 nm and its aggregation formed by several or more bundles of layers. Gu et al. [10] prepared nanocomposites involving styrene–butadiene rubber, natural rubber (NR), and organo-bentonite by emulsion interaction, and it was revealed that only nanocomposites with 12 wt% organo-bentonite showed the highest tensile strength (5.76 MPa), which was about 2.12 times higher than that of a virgin SBR/NR blend. Vishvanathperumal and Gopalakannan [16] prepared EPDM/SBR/Cloisite 30B nanocomposites using a two-cylinder mixer and it was revealed that the incorporation of nanoclay into the EPDM/SBR matrix improved the mechanical properties of SBR. Chakraborty et al. [17] used naturally °Ccurring unfractionated bentonite clay to prepare an SBR/bentonite nanocomposite by latex stage blending, and the hybrid material obtained showed that the interplanar distance of the in situ resol-modified bentonite clay increased from 1.23 to 1.41 nm for the modified bentonite and partial exfoliation and/or intercalation. Montmorillonite (MMT) was structurally modified by Zha et al. [18], who used the acid treatment followed by chemical modification of a γ-methacryloxy propyl trimethoxysilane molecule (KH570). It was found that the functionalization process changed the highly ordered stacking structure of the mineral MMT into a highly disordered structure. As can be concluded from these investigations, the properties of SBR combined with those of nano-organoclays as a hybrid material are promising in the development of selective membrane separation by pervaporation. Indeed, such a membrane combines excellent film-forming properties with good mechanical stability in many pure solvents or mixtures. Pervaporation, which is a membrane technique used in the selective separation of volatile organic–organic and organic–hydroorganic mixtures, is considered to be clean, cost effective, energy efficient, and easy to integrate into the refining chain of the chemical and petrochemical industry compared to conventional processes, such as distillation in its various forms [19,20,21,22,23,24]. Thus, the work of Singha et al. [25] on the removal of pyridine from its pyridine/water mixture using efficiently crosslinked SBR membranes using a sulfur/accelerator ratio less than unity and carbon black filler, and the results obtained showed better selectivity and mechanical strength but a lower total flux than the unloaded membrane. Recently, Mahapatra et al. [26] employed semi-efficiently vulcanized SBR membranes possessing an optimum balance between tensile strength and elongation at break and other composites involving carbon black as a filler in different compositions to optimize the separation of the tetrahydrofuran/water mixture by pervaporation. The results obtained were satisfactory.

Alcohol-aromatic mixtures such as a toluene–methanol system are commonly used in the petrochemical and pharmaceutical industries. This system forms an azeotropic with 32 wt% toluene at a constant pressure of 101.3 kPa [27]. Over the past two decades, the separation of such a mixture has attracted the particular attention of researchers in different fields due to the importance of the components in the chemical and pharmaceutical industries [28,29,30,31,32,33,34,35,36,37,38,39]. Different methods were used to solve the azeotrope problem, but the pervaporation technique was the most competitive for the reasons mentioned above. Indeed, to have better compromises between flux and selectivity, different membranes have been involved in this technique. Pol(vinyl alcohol) [28], cellulose triacetate, cellophane [29], polyimide [30], Y-zeolite [31] and others involving copolymers, such as cellulose triacetate–acrylic acid [29] and acrylonitrile–hydroxy ethyl methacrylate [30], and polymer blends, such as poly(acrylic acid)/poly(vinyl alcohol) [32], cellulose acetate/cellulose acetate butyrate [30], and poly(acrylic acid)/poly(vinyl alcohol) [32], have been developed in the separation of toluene/methanol mixtures. These materials all exhibited higher affinity toward methanol. As a reminder, the toluene–methanol azeotropic mixture contains less toluene (32 wt%), thus it is reasonable to consider a toluene selective membrane as it is easier to remove any component to achieve the separation goal. A very limited number of investigations have been reported in the literature on the development of toluene-selective membranes for such separation. Poly(dimethylsiloxane) (PDMS) [36], linear low density polyethylene [30], natural rubber (NR) based membranes [33,34], chitosan/polytetrafluoroethylene blend [35], crosslinked ethylene propylene diene monomer (EPDM) [36], poly(vinyl alcohol) [28], polyphenylene isophthalamide (PA) [37], polyurethane (PU)–poly(dimethylsiloxane) (PDMS) blend [38], styrene butadiene rubber (SBR), and multiwalled carbon nanotubes (MWCNT) incorporated styrene butadiene rubber (SBR) [34,39] are among the rare membrane materials reported to selectively extract toluene from the methanol/toluene mixture.

In this work, a series of crosslinked poly(styrene-co-butadiene) rubber/Maghnia organoclay montmorillonite (CSBR/OMMT) membranes containing different OMMT contents were prepared by a solvent casting method. To achieve this goal, organomontmonrillonite or organophylic clay (OMMT) as a filler was synthesized according to the procedure described by Bhattacharya and Aadhar [40]. The crosslinking reaction of SBR was carried out in situ in the presence of sulfur (crosslinker) and zinc diethyldithiocarbamate (catalyst). The hybrid materials obtained were characterized by FTIR, XRD, DSC and SEM analysis and the thermal, mechanical and the absorption properties of the prepared nanocomposites have been studied. As an application, the obtained CSBR/OMMT hybrid materials were used for the first time as pervaporation membranes to selectively separate the methanol/toluene azeotropic mixture. The effect of OMMT content in the CSBR/OMMT membrane on the fluxes (total and partial), and the separation factor was investigated. 

## 2. Experimental Section

### 2.1. Materials

Poly(styrene-co-butadiene) rubber with 45 wt% of styrene content and a density of 0.965 g·mL^−1^ was supplied by Sigma-Aldrich (Taufkirchen, Germany). Maghnia bentonite containing 98% montmorillonite [(Si_8_)^IV^ (Al_4−x_ Mg_x_)^VI^ O_20_(OH)_4_], having a cation exchange capacity (CEC) of 89 meq·g^−1^ was provided by “Entreprise Nationale des substances utiles et des produits non-ferreux”(ENOF) (Maghnia, Algeria). The chemical composition of this clay is presented in Table 1. The Cetyltrimethylammonium bromide (CTAB) (purity 99%) used as a modifier was purchased from Acros (Dallas, TX, USA). Zinc diethyldithiocarbamate (ZDC) was purchased from Chem Cruz of Santa Cruz Biotechnology (Dallas, TX, USA). Fine powder of elemental sulfur of a pale-yellow color was purchased from Fisher Scientific (Merelbeke, Belgium). Methanol (purity > 99) and toluene (purity > 99) were provided by AnalaR NORMAPUR (Darmstadt, Germany). Some physicochemical properties of the SBR, toluene and methanol are shown in Table 2. 

### 2.2. Synthesis of Organophilic Clay

Organomontmonrillonite or organophylic clay (OMMT) was synthesized according to the procedure described by Bhattacharya and Aadhar [40]. A total of 5 g of purified and dried bentonite was dispersed under vigorous stirring for 24 h in 500 ml of distilled water at room temperature (25 °C), and then 0.03 M of a CTAB solution was slowly added dropwise with continuous stirring for 12 h. The resulting mixture was then filtered under vacuum and the collected sediment was washed several times in distilled water and then dried under vacuum at 90 °C, crushed and sieved at 72 µm.

### 2.3. Preparation of CSBR/OMMT Nanocomposite

SBR solution was prepared by dissolution of small SBR pieces in toluene during 24 h. A determined amount of organoclay filler was then added in small portions under continuous stirring for 24 h to prepare a suspension. Sulfur (crosslinker) and ZDC (catalyst) were added to the previous mixture in a ratio of 1.5: 6 by weight, and the whole was left to stir for an additional 8 h. The contents were then carefully poured onto a horizontal glass plate and allowed to air dry for 12 h. Crosslinking of the SBR was then carried out in the presence of sulfur and ZDC by heating at 110 °C for 1 h. The cured thin film deposited on the glass plate was allowed to cool to room temperature then tromped into distilled water which was easily peeled off [33]. Different CSBR/OMMT membranes containing 0, 3, 6, 9, 12 and 15 parts by 1 hundred parts of OMMT in CSBR rubber (phr) were prepared by this same method and the experimental conditions are gathered in Table 3. The membrane thickness was then measured in different regions with a digital micrometer and the average thickness of the membranes used in the PV study was about 50 µm.

### 2.4. Membrane Characterisation

FTIR spectra were recorded on a Perkin Elmer Spectrum BX LX 185255 instrument with a scanning range of 400–4000 cm^−1^ under transmittance mode at 4 cm^−1^ increments. XRD patterns were obtained by a Siemens D-5000 X-ray diffractometer (30 kV, 10 mA) with Co (wavelength λ = 1.788965 Å) irradiation at a scanning rate of 0.02°/min in the range of 2–30°. SEM images were performed on films coated with gold grid using JSM-6060LV (JEOL). The particle size of the OMMT filler was measured by dynamic light scattering (DLS) (Malvern Instrument Ltd., London, UK) at room temperature (25 °C) after dilution of the formulations with double-distilled water. The value of the particle size was taken from the arithmetic mean of three replicates. The surface morphologies of OMMT, CSBR particles and CSBR/OMMT hybrid materials were studied by transmission electron microscopy (TEM JEOL JEM-2100 F) (Tokyo, Japan) at an acceleration voltage of 200 kV. The DSC thermograms were carried out on a DSC Q1000 (TA Instruments, Mississauga, ON, Canada) previously calibrated with indium. A total of 6–10 mg of film sample was packaged in a DSC aluminum pan before being placed in the DSC cell and heated under nitrogen gas from −80 to −10 °C at a heating rate of 20 °C min^−1^. TGA measurements of the samples were carried out under nitrogen gas on an SDT 600 device (TA Instruments, Mississauga, ON, Canada). A total of 4–10 mg of sample film was placed in an aluminum TGA pan and heated from 0 to 700 °C at a heating rate of 20 °C min^−1^. Mechanical strength measurements were performed on an Instron Zwick/Roell Z010 (Ulm, Germany) instrument at a heating rate of 0.3 mm·s^−1^. Tensile properties, such as tensile strength, 100% modulus and elongation at break were evaluated according to the norm DIN EN ISO 527-1. The crosslinking densities (*v*) for the pure CSBR and CSBR/AOMMT nanocomposites were estimated by a toluene swelling test. The film sample was immersed in an excess of toluene at room temperature (25 °C) until constant mass (equilibrium). The value of the crosslinking density was calculated using the Flory–Rehner Equation [43]. 

### 2.5. Swelling Experiments

Membrane swelling measurements in the azeotropic methanol/toluene mixture (32 wt% toluene) and their components were taken at 25 °C. Three square pieces of dried membrane of dimensions 4.0 cm × 4.0 cm × 1.2 ± 0.4 mm were weighed accurately and then immersed separately in the mixture and in its pure components, then stirred continuously until the swelling equilibrium was reached (constant mass). During this step, each film is removed from the container at determined time intervals. The swollen films are then gently wiped with tissue paper to remove liquid droplets deposited on both sides, then weighed on a precision balance. This experiment was repeated twice, and the results were obtained from the arithmetic mean of the two recorded weights. The degree of swelling (DS) was determined using Equation (1):

(1) $$S\left(\%\right)=\frac{w_{t}-w_{o}}{w_{o}}\times 100$$

where $w_{t}$ and $w_{o}$ are the weights of the swollen membrane at time *t* and the dried membrane, respectively.

### 2.6. Pervaporation Experiments

#### 2.6.1. Pervaporation Setup

**Scheme 2:**
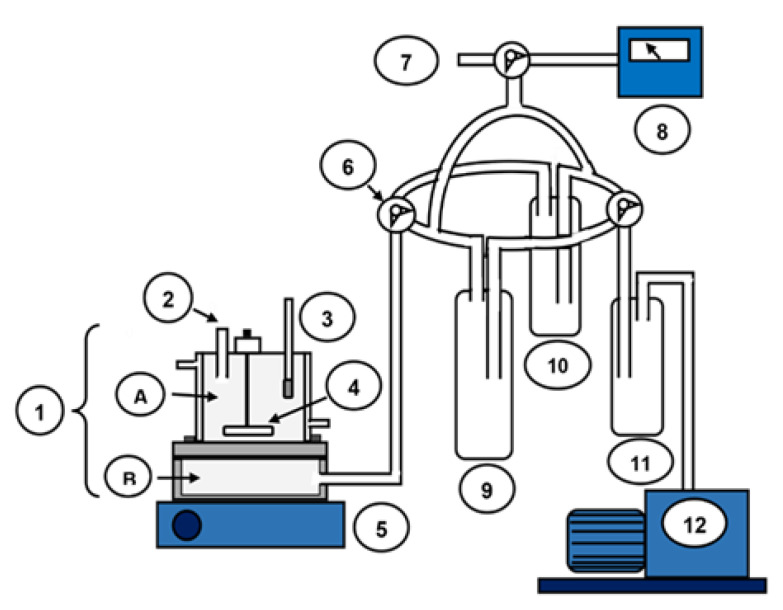
Pervaporation apparatus used in this investigation. **1**: pervaporation cell; **2**: feed inlet; **3**: thermometer; **4**: hanged magnetic bar; **5**: magnetic stirrer; **6**: three-way rotaflow valve; **7**: opening to the air; **8**: Pirani gauge; **9**: cold trap; **10**: cold trap; **11**: protection trap; **12**: vacuum pump.

The pervaporation apparatus used in this study (Scheme 2) is inspired by the literature [44,45,46]. The pervaporation cell (1) is made of stainless steel with a capacity of 125 cm^3^; it is divided into two compartments separated by a polymeric membrane deposited on a support containing interconnected pores made of the same metal. The effective surface area of the membrane in contact with the liquid feed is 29.28 cm^2^. The feed in the upstream compartment (A) of the membrane maintained at a known temperature is regularly homogenized using a suspended magnetic bar (4). The downstream compartment (B) through which the pervaporate passes is directly linked to the two siphon permeation traps (9) and (10) which operate alternately. The latter are immersed in liquid nitrogen and kept under vacuum (less than 1.6 mbar) using a vacuum pump (12) Vacuubrand PC 3001 VARIO Pro. The permeate obtained is analyzed by gas chromatography using a chromatograph SCION 436-GC equipped with a flame ionization detector (FID) heated to 270 °C and a Restek Rxi-5ms capillary column (30 m 0.32 m × 0.25 µm) heated to 120 °C. Helium was used as the carrier gas and the temperature of the injection port was 250 °C. 

#### 2.6.2. Pervaporative Parameters

The performance of the membrane is estimated by the permeation flux, J (g m^2^ h^−1^), and the separation factor, α, calculated using Equations (2) and (3) [33], respectively:

(2) $$J\;\left(gm^{-2}h^{-1}\right)=\frac{w}{tA}$$

where *w* (g) is the mass of the permeate collected during a time *t* (h) and *A* is the membrane surface area (m^2^):

(3) $$\alpha_{PV}=\frac{Y_{toluene}/Y_{methanol}}{X_{toluene}/X_{methanol}}$$

where $X_{toluene}$ and $X_{methanol}$ are the weight fractions of toluene and methanol in the feed, respectively. $X_{toluene}$ and $X_{methanol}$ are the weight fractions of toluene and methanol in the permeate.

The flux of each component calculated from the total flux obtained in the separation of the azeotropic mixture is calculated using Equation (4) [39]:

(4) $$J_{toluene\;}=JP_{toluene}\;\mathrm{and}\;J_{methanol\;}=JP_{methanol}$$

where $J_{toluene\;}$ and $J_{\mathrm{methanol}\;}$ are the contribution of the toluene and methanol in the total flux, *J*. 

The membrane efficiency was estimated using the pervaporation separation index, PSI, calculated using Equation (5) [47]:

(5) $$PSI=J\times (\alpha_{PV}-1)$$

The apparent diffusion coefficient, $D_{i}\left(m^{2}\cdot s^{-1}\right)$, was calculated from the modified Fick’s Equation (6) [48] and the data of the transmembranar flux of each component:

(6) $$D_{i}=\frac{J_{i}\times d}{C_{i}}$$

where J , *C* and *d* are the flux (kg·m^−2^·s^−1^), the concentration of the permeate (kg·m^−3^) and the membrane thickness (m), respectively. The subscript *i* stands for toluene or methanol.

## 3. Results and Discussion

### 3.1. Membrane Characterization

#### 3.1.1. FTIR Analysis

**Figure 1:**
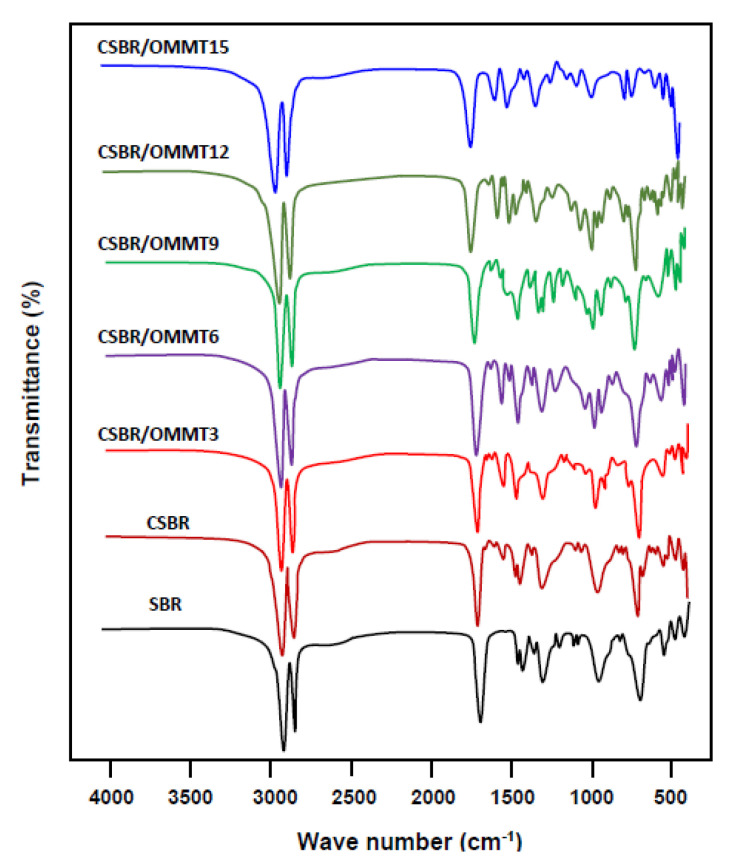
FTIR spectra of SBR, CSBR and CSBR/OMMT nanocomposites with different OMMT contents.

Figure 1 shows FTIR spectra of pure SBR, CSBR and CSBR/OMMT nanocomposites containing different OMMT contents. These traces show strong absorption bands in the range of 2920–2850 cm^−1^ characterizing the asymmetric and symmetric stretching frequency of the -C-H, -C-CH_3_ and -CH_2_- groups. The absorption bands observed at 1444 and 1301 cm^−1^ are attributed to the plane deformation of the -CH_2_ bond and its stretching motion, respectively. The stretching of the C=C double bonds in *trans* is observed at 961 cm^−1^ [49]. The stretching vibrations of C=C double bonds of the benzene ring are located at 1434, 1464 and 1696 cm^−1^, while the absorption bands at 694 and 758 cm^−1^ are assigned to the substituent benzene ring of styryl unit [50]. The spectrum of CSBR shows all its characteristic absorption signals, including that due to the stretching and bending of the bonds formed by the crosslinking reactions. Comparison of this spectrum with that of the pure SBR film reveals the appearance of an additional absorption band at ~777 cm^−1^ which is attributed to the carbon–sulfur bond (CS). This is due to the stretching of the monosulfide bond (total crosslinking). The low absorption band observed at ~538 cm^−1^ is attributed to the stretching of the sulfur–sulfur bond (SS) [33,34]. The spectrum of the CSBR/OMMT3 hybrid material does not show additional information. Due to the low loads of OMMT added, the spectrum shows no significant characteristic signal or peak shift belonging to the clay or its hydrogen bond-like interaction with the polymer. Such an observation has also been reported in the literature [51].

#### 3.1.2. XRD Analysis

**Figure 2:**
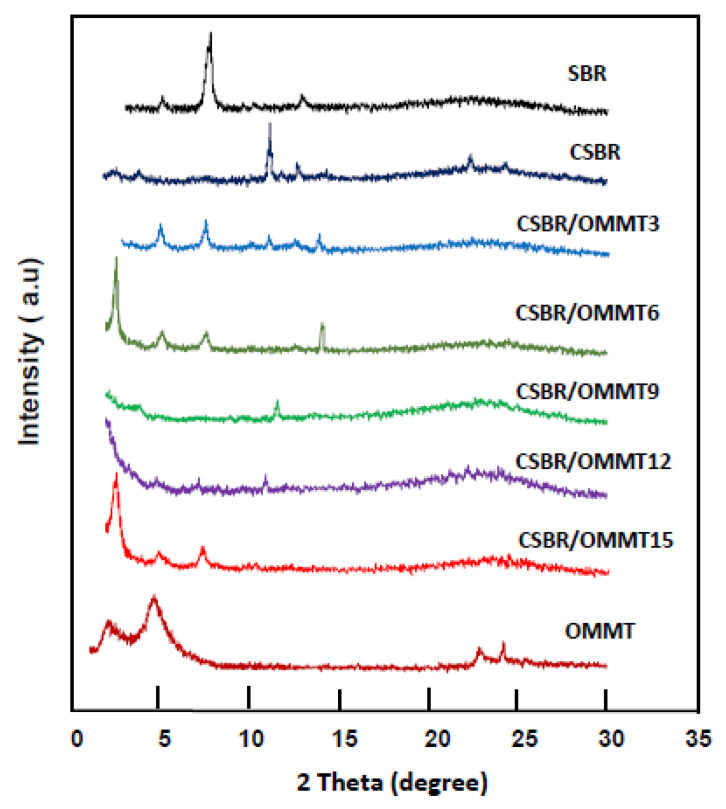
XRD spectra of OMMT, SBR, CSBR, and CSBR/OMMT nanocomposites with different OMMT contents.

The X-ray diffraction spectra of the CSBR/OMMT nanocomposite containing 3, 6, 9, 12 and 15 phr of OMMT are shown in Figure 2. The XRD pattern of OMMT shows a large peak at 2θ = 4.231°, which corresponds to a basal spacing (001) of 24.227 Å, calculated using the Bragg Equation (7) [39]:

(7) $$\lambda =\frac{2d\text{sin}\theta }{n}$$

where λ and *d* are the wavelength of the X-ray and the spacing of the crystal layers (path difference). The incident angle is θ (the angle between incident ray and the scatter plane), and *n* is an integer.

These values indicate that the OMMT layers are widely separated by cetyltrimethylammonium ions [52]. Signals belonging to CSBR/OMMT hybrid materials show typical behavior of an amorphous polymer through its picks at 2.63°, 5.17°, 7.75°, 14.46°, and 22.61° 2 theta (intense) with a d spacing of 38.83, 19.82, 13.22, 17.10 and 4.55, respectively. The disappearance of the diffraction peaks in the OMMT layers suggests that this clay has been exfoliated and dispersed in the polymer matrix to form a nanocomposite structure. This appears to be due to van der Waals-type interactions between the long carbon chains of the modified clay and the nonpolar rubber chains. The latter seems to have better interactions with organically modified clay, compared to those of unmodified clay, due to better surface compatibility [53]. 

#### 3.1.3. SEM Analysis

**Figure 3:**
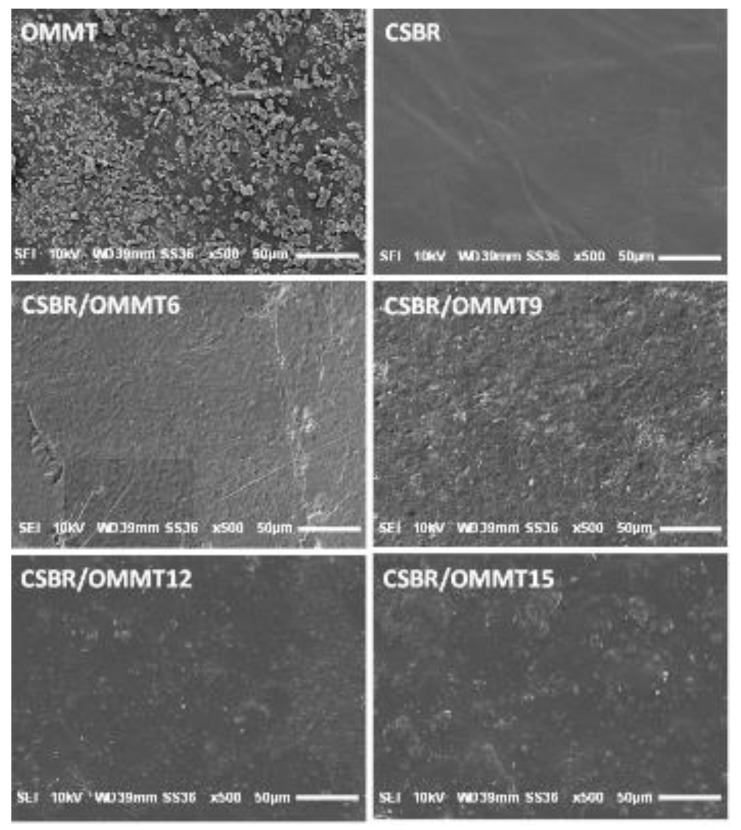
SEM micrographs of: (A) OMMT nanoparticles; (B) CSBR; (C) CSBR/OMMT6; (D) CSBR/OMMT9; (E) CSBR/OMMT12; (F) CSBR/OMMT15 systems.

SEM micrographs of OMMT powder, CSBR films and CSBR/OMMT hybrid materials are shown in Figure 3. The image of pure OMMT shows aggregated nanoparticles in different forms. The particle size of the aggregates as illustrated in this image is between 0.52 and 76 μm. The CSBR image shows a smooth surface morphology devoid of any particles which may have originated from solid residues of ZDC used as a catalyst in the polymer crosslinking reaction. The images of the CSBR/OMMT hybrid materials show, for all the compositions, a uniform dispersion of organic clay nanoparticles slightly recessed and covered with thin rubber film. The grouping of nanoparticles on these surfaces as it appears on these micrographs is probably due to their structure. Similar micrographs were also obtained by different authors using SBR [52], NR [52] and EPDM [53] membranes filled with N330 carbon black. The morphology of these materials became "coarser" as the filling load increased. SEM micrographs of the hybrid materials (films) and their components (CSBR film and OMMT powder) are shown in Figure 3.

#### 3.1.4. DLS Analysis

**Figure 4:**
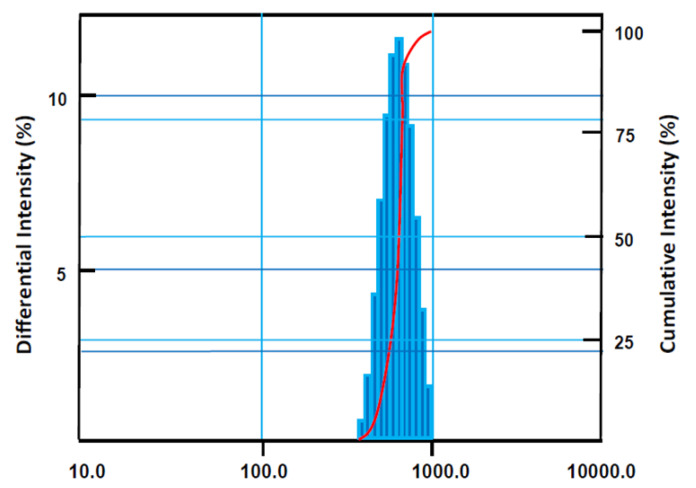
Histogram of the particle size distribution and accumulated fraction of OMMT nanoparticles.

Figure 4 shows the histogram of the particle size distribution and the accumulated fraction of OMMT nanoparticles obtained by the DLS method. This analysis revealed 654 nm as the mean diameter of the organofiller sample and 0.446 as the polydispersity index.

#### 3.1.5. DSC Analysis

**Figure 5:**
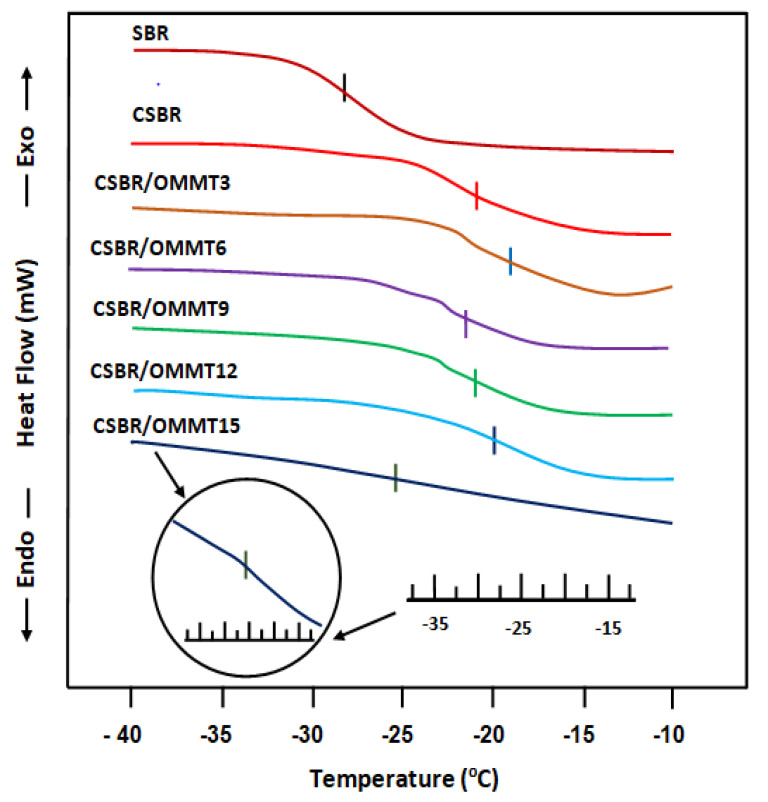
DSC thermograms of pure SBR, CSBR and CSBR/OMMT nanocomposites.

The DSC thermograms of pure SBR, CSBR, and CSBR/OMMT hybrid materials containing different OMMT contents are gathered in Figure 5 and the values of the glass transition temperatures, *Tgs*, deducted from these curve profiles are gathered for comparison in Table 4. The *Tg* value of pure SBR is localized at −27.8 °C, which agrees with that containing similar styrene/butadiene composition (45%) in the literature [54]. When this copolymer was crosslinked (CSBR), its *Tg* shifted significantly to the higher temperatures (from −27.8 to −21.5 °C). This is mainly due to a significant reduction in chain sliding caused by the crosslinking of the polymer, in which the greater the degree of crosslinking of the polymer increases, the more the chain sliding is reduced and the higher the value of *Tg* is [55]. When the OMMT nanoparticles were incorporated in the CSBR matrix, the *Tg* shifted toward higher temperatures for the CSBR/OMMT3. This can be explained by the adhesion of the OMMT nanoparticles to CSBR due to relatively weak attractive interactions between the two components at this composition [56,57,58,59,60,61]. The weak interactions between the CSBR and the OMMT are due to the weak polarity of the aromatic ring of the styryl group of the CSBR and those of the metals of the oxides contained in the OMMT. On the other hand, when the nanofiller was at more than 3 wt% in the hybrid material, the excess of OMMT played the role of rollers, facilitating the sliding of the chains and thus leading to lower *Tg*. In this case the value of *Tg* drops from −20 °C for the CSBR/OMMT3 to −25.5 °C for the CSBR/OMMT15, except that of the CSBR/OMMT12, where the *Tg* remains practically unchanged. This seems to be due to a phenomenon of compensatory equilibrium between the polymer–OMMT adhesion which takes place at a maximum load of 3% (shift towards high temperatures) and the chain sliding which takes place at higher loads (shift towards low temperatures). Similar explanations have also been reported by different authors using other hybrid systems [62].

#### 3.1.6. TGA/DTG Analysis

**Figure 6:**
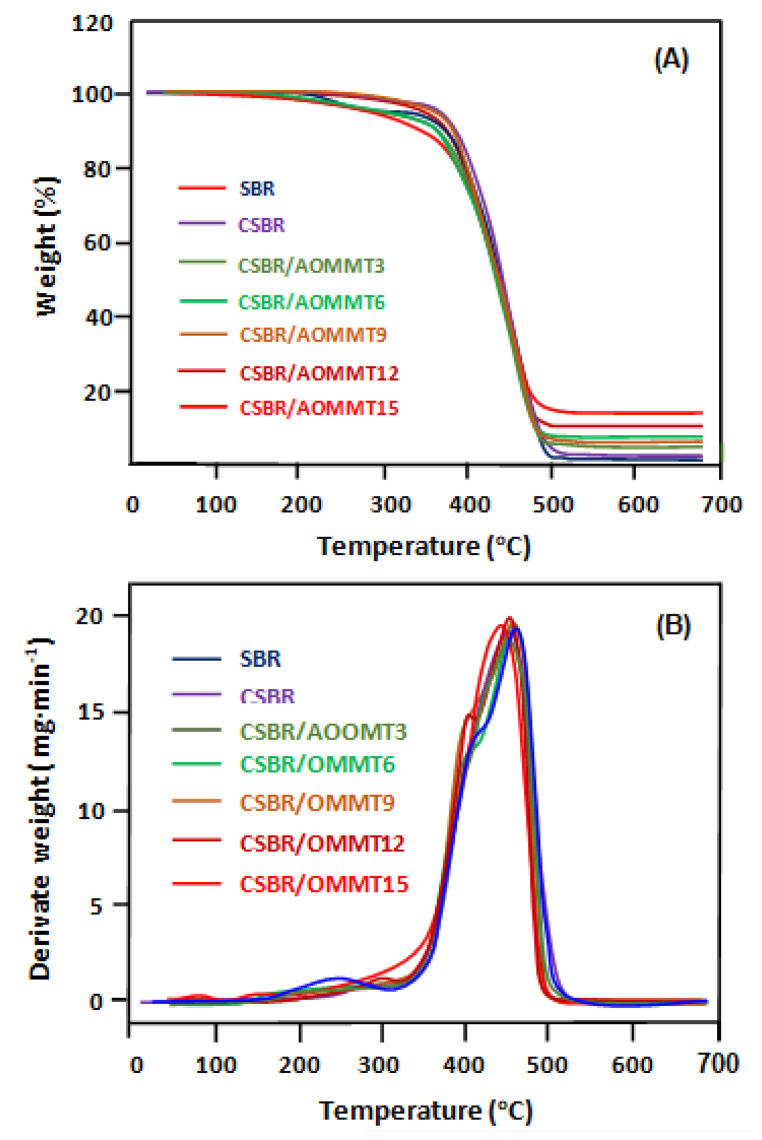
TGA (**A**) and DTG (**B**) thermograms of SBR, CSBR and CSBR/OMMT nanocomposites with different OMMT contents.

TGA and DTG thermograms of pure SBR, CSBR and CSBR/OMMT nanocomposites with different OMMT contents are gathered for comparison in Figure 6A,B, respectively. As can be observed from these curve profiles, all samples show two decomposition temperatures, *Tds*, which are more marked in the thermal curves of DTG (Figure 6B). The first decomposition temperature of pure SBR begins at 350 °C with a weight loss of 6.4% and the second at about 400 °C with a weight loss of 10%. The thermogram of the crosslinked SBR (CSBR) shows a reduction in its thermal stability of 5 °C, in which 4.1 wt% of its mass is released. The incorporation of OMMT into the rubber matrix did not significantly affect the thermal stability of this copolymer, but accelerated its decomposition process as shown on the DTG thermograms of Figure 6B. For example, at a temperature of 350 °C, CSBR loses 4.1 wt% of its initial mass, while those of nanocomposites containing 3, 6, 9, 12 and 15 phr lose 6, 8, 3, 5 and 10 wt%, respectively. The second decomposition temperature begins, for all samples, at about 400 °C, at which between 9.8 and 11.0 wt% of the SBR is released. On the other hand, as shown by the DTG thermal curves (Figure 6B), the maximum decomposition rate fluctuates randomly between 19 and 19.6 mg·min^−1^ for all the nanocomposites except the one containing 15 phr of OMMT, which was of 17.0 mg·min^−1^.

The slowing of the maximum rate of decomposition observed in the case of the hybrid material containing 15 phr of OMMT is probably due to additional absorption energy created by a large slippage of the SBR chains. The large sliding of the copolymer chains is favored by the large spacing caused by the excess of aggregates of nanofillers encrusted between the SBR chains. This phenomenon was also demonstrated previously through the results of DSC through the sudden shift of *Tg* towards the low temperatures.

### 3.2. Mechanical Strength

The mechanical properties of CSBR nanocomposites reinforced with OMMT filler, such as tensile strength, elongation at break, and 100% modulus of elongation, are shown in Table 5. These results revealed that an increase in OMMT loading in the CSBR rubber matrix results in a linear increase in mechanical properties. Comparable results were also obtained by El-Sabbagh et al. [8,63] and Noriman et al. [8,63] using mixtures of styrene/butadiene/modified clay, styrene/butadiene, styrene/butadiene, and recycled acrylonitrile/butadiene. The tensile strength of the CSBR filled with 15 phr of OMMT was about twice that of the cured CSBR, and the elongation at the break increased with increasing OMMT loading. In particular, an increase of 210% was obtained for the nanocomposites containing 15 phr of OMMT. This can be explained by the interlaced/exfoliated structure of the loaded organo-clay SBR vulcanizates, which allows the silicate layers to orient in the direction of the stress. It also helps to increase tensile strength and elongation at the break. The increase in the degree of chemical and physical crosslinking and the rate of slip of the copolymer chains on the surface and between the silicate layers can also cause an increase in the elongation at the break. Such a phenomenon has also been reported separately by Diez et al. [64] and Choi et al. [65]. The effect of the CSBR reinforcement is estimated based on the values of the modulus at 100% elongation (M100), which is also considered as an indicator of the stiffness of rubbers. The increase in the modulus is obtained by the dispersion of the silicate layers in the copolymer matrix. This ensures an intense interaction between the two components due to the interactions created between the silicate layers and the polymer matrix. Indeed, the strong interaction between the chains of the copolymer and the surface of the clay favors the promotion of Young’s modulus. According to El-Sabbagh et al. [8,63] on the one hand and Helaly et al. [8,66] on the other hand, the mobility of rubber chains is retarded near the surface of the organobentonite (OB). It was also revealed that M100 increased slightly with the addition of an organic clay filler and the maximum improvement was achieved at a 15 wt% load.

### 3.3. Crosslink Density

Crosslinking reactions are important in improving the overall performance of rubber and efficient vulcanization results in maximum monosulfide bonding [67,68]. Here, the crosslinking densities of SBR compounds with various filler contents were evaluated using the Flory–Rehner equation [43]. Indeed, the crosslink density of CSBR has been quantitatively estimated through the equilibrium swelling using Equation (8):

(8) $$\upsilon =-\frac{1}{V_{s}}\;\left[\frac{\ln\left(1-v_{r}\right)+v_{r}+\chi v_{r}^{2}}{v_{r}^{1/3}-\frac{1}{2}v_{r}}\right]$$

where v is the crosslink density (mol·cm^−3^), $V_{s}$ is the molar volume of solvent (106.2 cm^3^·mol^−1^ for toluene), and $v_{r}$ is the volume fraction of the crosslinked polymer swollen to equilibrium. χ is the Flory–Huggins interaction parameter between toluene and SBR (0.31) [69]. The value of $v_{r}$ is obtained according to Equation (9):

(9) $$v_{r}=\frac{w_{1}/\;\rho_{1}}{w_{1}/\;\rho_{1}+w_{2}/\rho_{2}}$$

where $w_{1}$ and *w_2_* are the weight of dry rubber and the swollen rubber, respectively. $\rho_{1}$ and $\;\rho_{2}$ are the densities of the rubber and the solvent, respectively. The values of the crosslinking densities obtained are given in Table 6.

**Figure 7:**
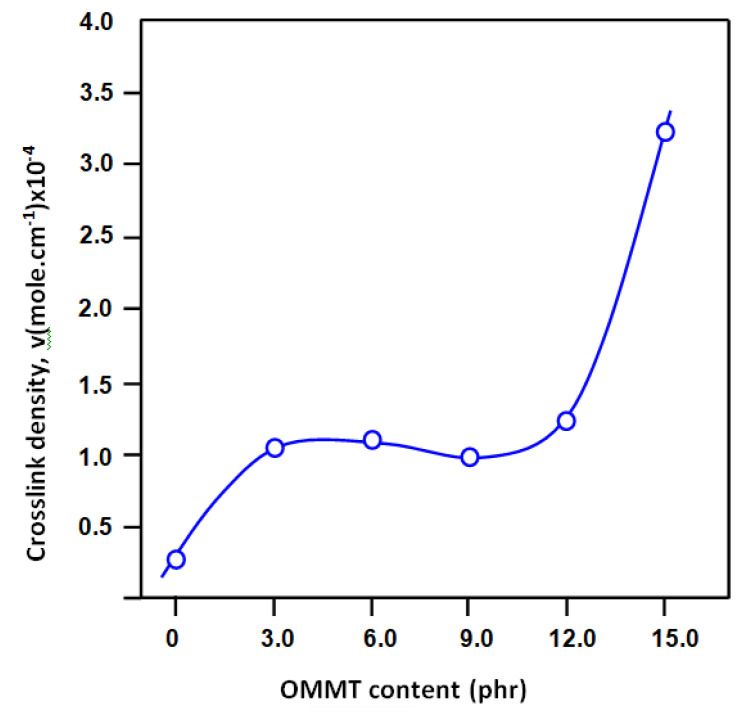
Variation of crosslink density of CSBR versus the OMMT content in the CSBR/OMMT membrane.

The variation of the crosslinking density of the CSBR/OMMT material as a function of the OMMT content is shown in Figure 7. As shown in the profile of the curve obtained, the crosslinking density of the hybrid material increases with the OMMT content and reaches a pseudo-stability at 1.05 ± 0.06 × 10^4^ mol·cm^−1^ between 3 and 9 phr, then continues to increase beyond. The increase in the *v* value seems to be due to the fact that a part of the space between the SBR chains fills up with the incorporated OMMT nanoparticles, thus limiting the amount of toluene absorbed. The pseudo-stability of the *v* value observed between 3 and 9 phr in OMMT content seems to be due mainly to an equilibrium established between the affinity of toluene with respect to the copolymer favoring the increase in the degree of swelling of CSBR and, on the other hand, the incorporation of more nanofillers decreases the number of the mixture-membrane contacts, which promotes the swelling capacity of the membrane. Note that a high crosslink density reduces the swelling capacity of the polymer.

### 3.4. Swelling Performance

**Figure 8:**
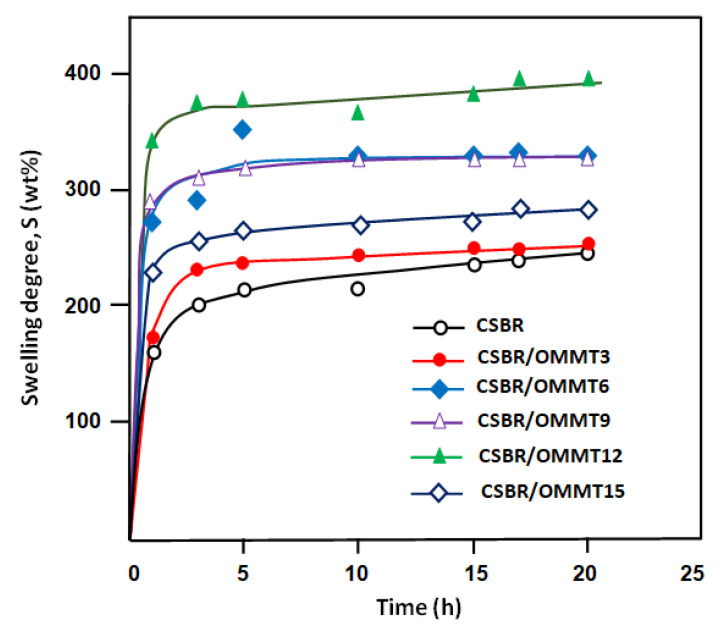
Variation of the swelling degree of CSBR and CSBR/OMMT membranes with different OMMT contents versus time in toluene.

**Figure 9:**
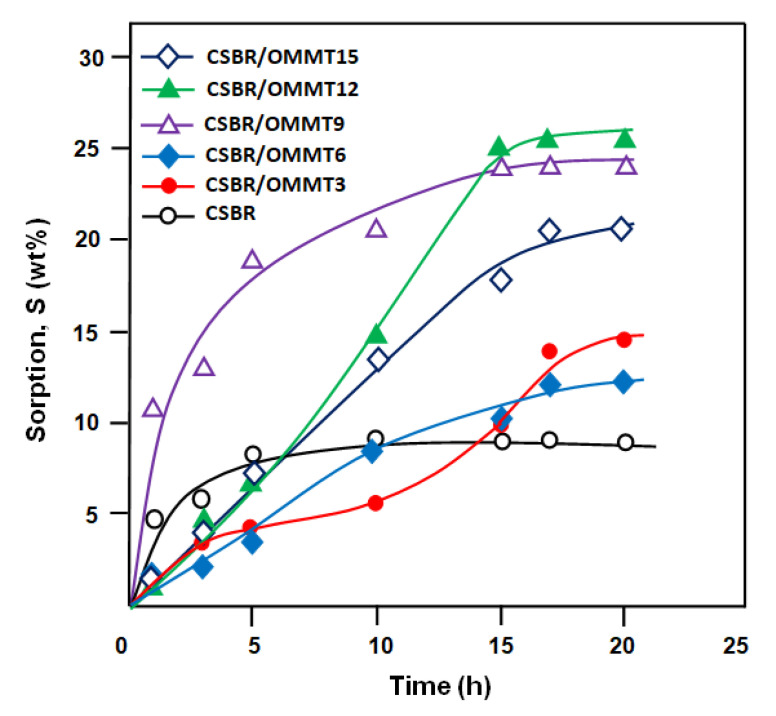
Variation of swelling degree of CSBR and CSBR/OMMT membranes with different OMMT contents versus time in methanol.

**Figure 10:**
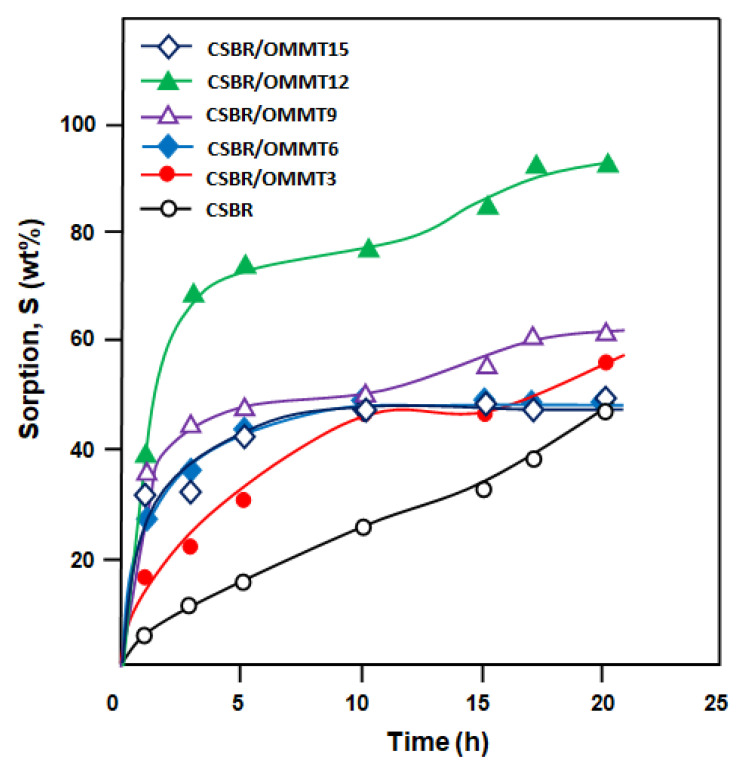
Variation of swelling degree of CSBR and CSBR/OMMT membranes with different OMMT contents in the azeotropic mixture versus time.

The results of the swelling degree for all membranes in the azeotropic methanol/toluene mixture and in their pure components as a function of time are grouped in Figure 8, Figure 9 and Figure 10. As can be seen from the curve profiles in Figure 8, the swelling capacity of the virgin CSBR membrane in toluene reached 242 wt% during 20 h of the swelling process. On the other hand, the incorporation of OMMT nanoparticles into the CSBR matrix significantly increases the selling degree of the resulting membrane. Indeed, it was revealed that the maximum absorption of toluene was reached with the CSBR/OMMT membrane containing 12 phr of OMMT content, in which 390 wt% of its mass was absorbed during this same period. Figure 9 shows the variation in the swelling degree of these same membranes in methanol as a function of time. The profiles of the curves obtained reveal that the swelling capacity of the virgin CSBR membrane in methanol was 9% by weight of its mass, which is much lower than that of this membrane in toluene during the same period. This seems to be obvious if one takes into account the large difference between the affinity of the membrane toward each component. Indeed, the difference between the Hansen solubility parameters of the SBR-toluene system $[\delta_{\mathrm{SBR}}-\delta_{\mathrm{Toluene}}=1.70\;$(J·cm^−3^)^0.5^] is much lower compared to that of the SBR–Methanol system $[\delta_{\mathrm{SBR}}-\delta_{\mathrm{Toluene}}$ = 13.10 (J·cm^−3^)^0.5^]. According to the Hildelbrand and Scott Equation (10) [70], for a higher solubility of a given polymer in a solvent, the value of the enthalpy of solvent–polymer mixture (Δ*H_M_*) must be very low (close to zero):

(10) $$\Delta H_{M}=V{\left(\delta_{1}-\delta_{2}\right)}^{2}\varphi_{1}\varphi_{2}$$

where $\delta_{1}$ and $\delta_{2}$ are the Hansen solubility parameters of solvent and polymer, respectively. $\varphi_{1}$ and $\varphi_{2}$ are the volume fractions of solvent and polymer, respectively, and *V* is the total volume of the polymer/solvent mixture. 

As shown by the profiles of the curves in the Figure 8, Figure 9 and Figure 10, the order of the swelling capacity of the membrane by each component and their azeotropic mixture as a function of the OMMT content is random. This may be due to two main competitive factors acting simultaneously on the swelling dynamics of the membrane. These two factors are interactional, such as the affinity between the different components of the solvent–SBR, solvent–OMMT and SBR–OMMT types, and morphological, such as the volume of the space between the chains of copolymers. For the toluene–membrane system, (Figure 8), the incorporation of more OMMT nanoparticles into the CSBR matrix promotes the increase in the free volume between the chains of the copolymer, thus allowing the insertion of more toluene molecules into the membrane matrix. On the other hand, the presence of more OMMT fillers between the polymer chains also reduces the contact surface between the toluene molecules and the polymer chains, and this goes in the direction of reducing the insertion capacity of toluene between the copolymer chains. This phenomenon is more marked when the OMMT content passed from 12 to 15 phr in the copolymer matrix in which the rate of swelling drops from 390 to 280% by weight. On the other hand, in the case of the membrane–methanol system (Figure 9), the increase in the percentage of OMMT nanofiller in the CSBR matrix leads to an increase in the number of OMMT–methanol surface contact to the detriment of that of CSBR–methanol. This goes in the direction of further promoting the development of interactions between these two chemical entities leading to the absorption of more methanol molecules by the membrane. For example, the incorporation of 9 phr and more of OMT in the CSBR matrix significantly increases the degree of swelling compared to those of 3 and 6 phr. 

In the case of the swelling of the membrane in the azeotropic mixture, Figure 10 shows a more complex situation. In addition to the two factors mentioned above, other parameters linked to the interactions between the two components of the mixture intervene to preserve the best performances of the CSBR/OMMT12 membrane. These factors lead to the convergence of all the other membranes towards similar degrees of swelling (47–60 wt%). Different studies reported in the literature involving toluene–methanol mixtures have shown similar trends for nanocomposites combining NR with carbon black [31], NR with bentonite clay [31], NR with zeolite [33] and SBR with carbon black [68]. 

### 3.5. Pervaporation Performance

**Figure 11:**
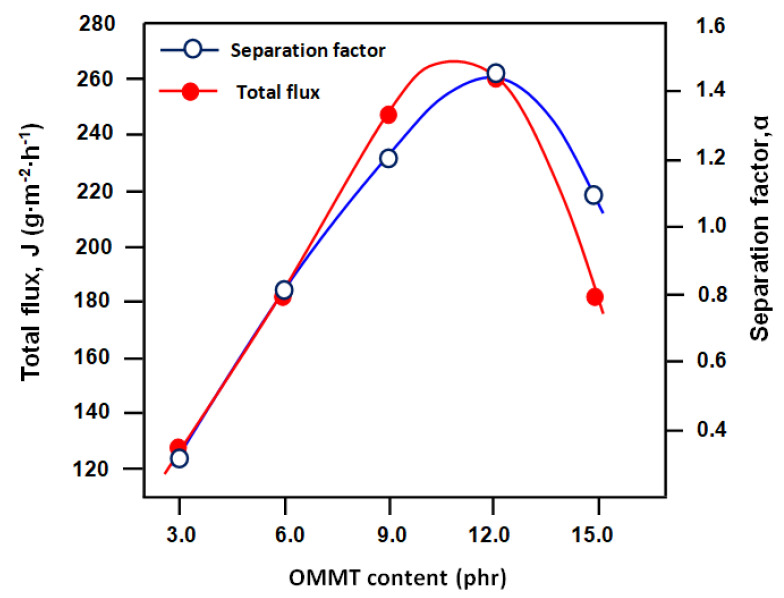
Variation in the trans-membranar flux and the separation factor versus the concentration of OMMT for the separation of the toluene–methanol azeotropic mixture.

In this section, a study is presented on the incorporation of OMMT nanoparticles into the vulcanized SBR membrane and its performance in terms of the pervaporative parameters in the separation of the azeotropic methanol/toluene mixture by the pervaporation technique. Figure 11 shows the effect of the OMMT/CSBR composition of the prepared membrane on the trans-membranar flux and the separation factor. As can be seen from the curve profiles obtained, the total flux increases with the OMMT content and reaches its maximum when the OMMT/CSBR composition is 12 phr. Whereas, when the clay content reaches 12 phr in the CSBR/OMMT membrane, the total permeation flux decreases significantly. These results are in the same direction as the swelling behavior of this membrane in the azeotropic mixture observed during the swelling experiments (Section 3.5). Indeed, it was observed that the CSBR/OMMT12 membrane absorbed the largest amount of toluene/methanol mixture. The explanation of this phenomenon in the case of swelling experiments also remains valid in that of the pervaporative flux, as long as the reasons are still present in the membrane.

The variation of the separation factor as a function of the OMMT load follows practically the same trend as that of the total flux with a slight shift of its maximum towards the higher compositions. This is predictable, as in the case of the comparison between the results of the trans-membranar flux and those of the swelling of the same membrane. Because of the swelling capacity of the membrane in pure toluene (Figure 8) and in pure methanol (Figure 9), it reveals that toluene is by far the most valued by the membrane used regardless of the composition of the membrane. However, besides the higher affinity between toluene and SBR compared to that between methanol and SBR, the transit of toluene molecules through the membrane is also faster regardless of the composition of the membrane. Therefore, the selectivity of the membrane towards toluene must follow the same path as that of the flux.

**Figure 12:**
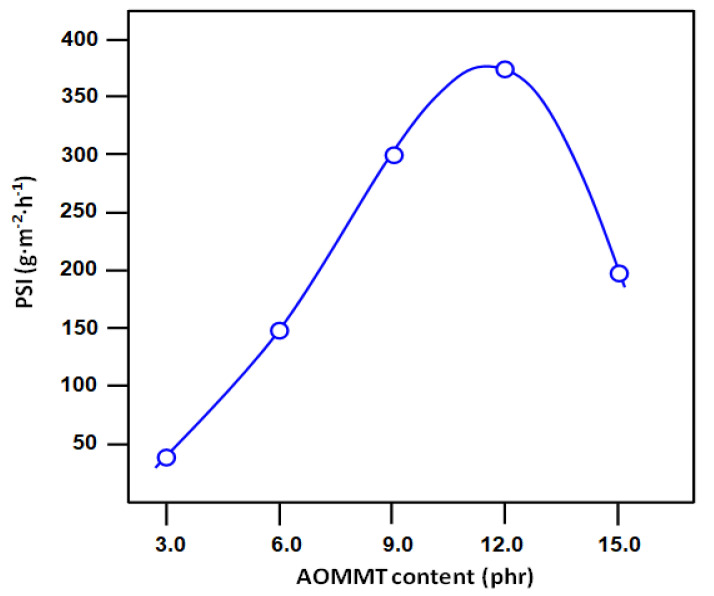
Variation in PSI versus the OMMT content in the OMMT/CSBR membrane for the separation of the azeotropic toluene/methanol mixture.

The Pervaporative Separation Index (PSI) provides insight into the performance of the membrane. In fact, the higher the PSI, the more efficient the membrane. Figure 12 shows the variation of PSI as a function of the OMMT content in the membrane and Table 7 groups together the PSI values calculated from Equation (5) and the data of Figure 11. The curve profile obtained was predicted, since the total flux and the separation factor varied with the filler content following the same trend and the best performance is obtained with the membrane containing 12 phr of OMT, in which the value of PSI reached 374.5 g m^−2^ h^−1^. These results indicate that the hybrid CSBR/OMMT12 material is well suited for use as a membrane in the separation of the toluene/methanol azeotropic mixture by the pervaporation technique. Table 8 shows the comparison of the results obtained in this present work with those of the literature using other rubbery membranes in the separation of toluene/methanol mixtures [30,33,34,38,70,71,72,73]. As can be seen from these data, the CSBR/OMMT12 membrane seems to be competitive with those in the literature, especially in terms of total transmembrane flux. Indeed, the performance of this membrane estimated from the PSI value ranges between that of NR-3 (55.2 g m^−^^2^ h^−^^1^) and that of PDMS (159.25 g m^−2^ h^−1^).

**Figure 13:**
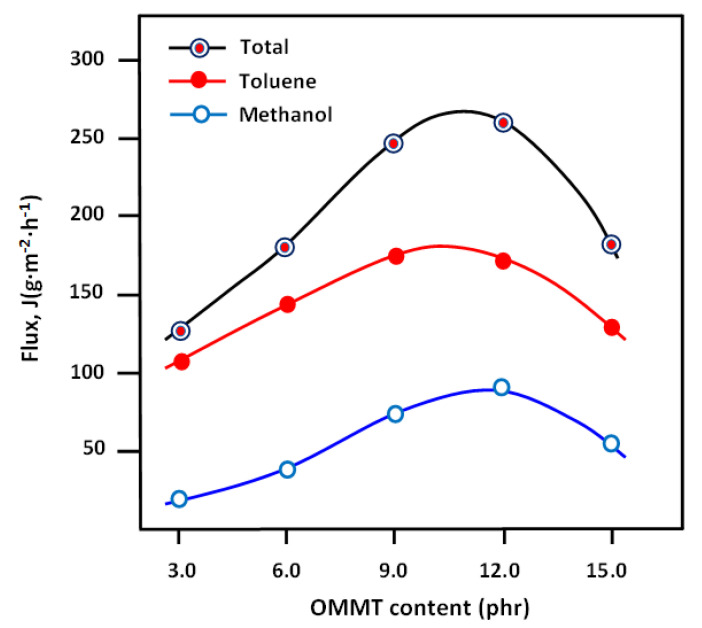
Variation in partial and total fluxes versus the OMMT content in the membrane for the separation of azeotropic toluene−methanol mixture.

Figure 13 displays a comparison of the variation of the total flux with those of toluene and methanol separately as a function of the OMMT content. These curves show similar profiles and confirm the explanations given previously on the selectivity results in the previous section. The flux of toluene is much greater than that of ethanol regardless of the percentage of OMMT in the membrane. These curve profiles show similar trends confirming the explanation given in the case of the results presented in this figure. It is evident that the flux of a component increases with the addition of 3 phr of OMT incorporated in the membrane. The total flux mainly contains toluene rather than methanol, as long as the membranes manufactured are selective for toluene.

The increased affinity of SBR/OMMT nanocomposite membranes for toluene is the driving force behind the remarkable difference in the separation of toluene from the methanol/toluene mixture [39]. Some reasons that can be noted are attributed to the separation performance. These reasons reside in the difference in the values of the solubility parameter (∆δ) of each component of the mixture to be separated from that of the membrane. The smaller the difference between the Hansen solubility parameter, the greater the degree of swelling. This is the case of the SBR-toluene system which shows ∆δ = 2.0 (J·cm^−3^)^0.5^ as much lower than that of SBR-methanol [∆δ = 13.2 (J·cm^−3^)^0.5^]. This further promotes the transport of toluene across the membrane. 

### 3.6. Diffusion

The mass transport of a binary liquid in a pervaporation membrane is usually explained by a solution diffusion mechanism, which takes place in three stages: sorption, diffusion, and evaporation. Thus, the rate of permeation and selectivity of the membrane are governed by the solubility and diffusivity of each component of the mixture. In the pervaporation technique, as long as the establishment of a distribution equilibrium is rapid between the feed and the upstream surface of a membrane, it is the diffusion step which controls the migration of the penetrant. Therefore, it is important to estimate the diffusion coefficient of penetrating molecules to understand the mechanism of the molecular transport. The diffusion coefficient is then an important kinetic parameter for estimating the diffusion of penetrants through a membrane. The *Di* values for the different CSBR/OMMT nanocomposite membranes are calculated from Equation (10) and the results obtained are collated in Table 9. 

As shown by the data obtained, the *Di* values of toluene are almost three times higher than those of methanol for all membranes. These results once again confirm the observation of the greatest sorption of toluene compared to that of methanol. This fact clearly explains the greater selectivity of CSBR/OMMT membranes towards toluene. 

## 4. Conclusions

Nanocomposite membranes involving organo-montmorillonite and SBR crosslinked in situ by an efficient vulcanization technique using sulfur as a crosslinking agent and zinc diethyldithiocarbamate as a catalyst have been successfully prepared. The uniform distribution of OMMT nanoparticles in the prepared CSBR/OMMT hybrid materials was confirmed by FTIR, XRD and SEM analysis. Thermal analysis of the resulting hybrid material performed by DSC revealed that the incorporation of OMMT nanoparticles into the CSBR matrix slightly improves the glass transition temperature of the pure SBR copolymer (Δ*Tg*, max = 7.8 °C) and retains its flexibility regardless of the OMMT content incorporated in the hybrid material. In fact, the *Tg* value of the CSBR/OMMT nacomposite reached a maximum of −20 °C, when the OMMT/CSBR composition was 3 phr. Thermal analysis performed by TGA/ATD reveals that there is virtually no noticeable change in the thermal stability of the prepared materials regardless of the composition of the composite system. The mechanical properties of the CSBR/OMMT hybrid material in terms of tensile strength, elongation at break and modulus at 100% elongation showed a remarkable increase as the OMMT content in the membrane increased. 

The application of the hybrid materials prepared as membranes for the separation of the toluene/methanol azeotropic mixture by pervaporation revealed that the total flux improved much better compared to those in the literature using membranes based on SBR and carbon black. The trans-membranar total flux follows the same trend as the partial fluxes attributed to each component of the mixture. These fluxes and separation factors increase with the OMMT content to reach a maximum at 12 phr of the load in the membrane. The behaviors of the total and partial fluxes follow practically the same trend as the swelling degree of the membrane in the azeotropic mixture. The maximum amount of toluene/methanol mixture was reached with the CSBR/OMMT12 membrane. Finally, this study reveals that the membrane containing 12 phr of OMMT nanoparticles makes it possible to obtain the best performance, particularly in terms of total flux. 

## Figures and Tables

**Table 1:** Chemical compositions of the Algerian montmorillonite used [41].

Species	SiO_2_	Al_2_O_3_	Fe_2_O_3_	MgO	CaO	Na_2_O	K_2_O	TiO_2_	As	L.O.I
Percentage (wt%)	69.4	14.7	1.2	1.1	0.3	0.5	0.8	0.2	0.05	11

**Table 2:** Properties of solvent and rubber [42].

Compound	Molar Volume (cm^3^ mol^−1^)	Molar Mass (g·mol^−1^)	Density (g·cm^−3^)	Vapor Pressure at 25 °C	δ * (J·cm^−3^)^0.5^
Methanol	40.4	32.04	0.792	94.0	29.7
Toluene	107.1	92.14	0.867	28.4	18.2
SBR	-	140,000	0.980	-	16.5

* Hansen solubility parameter.

**Table 3:** Experimental conditions of the preparation of CSBR/OMMT membranes.

Membrane	SBR (phr)	Sulfur (phr)	ZDC (phr)	OMMT (phr)
pure SBR	100	0	0	0
CSBR	100	1.5	6.0	0
CSBR/OMMT-3	100	1.5	6.0	3
CSBR/OMMT-6	100	1.5	6.0	6
CSBR/OMMT-9	100	1.5	6.0	9
CSBR/OMMT-12	100	1.5	6.0	12
CSBR/OMMT-15	100	1.5	6.0	15

**Table 4:** Glass transition temperatures of SBR and SBR/OMMT nanocomposites.

Membrane	Tg (°C)
SBR pure	−27.8
CSBR	−21.5
CSBR/OMMT3	−20.0
CSBR/OMMT6	−22.0
CSBR/OMMT9	−22.5
CSBR/OMMT12	−21.5
CSBR/OMMT15	−25.5

**Table 5:** Mechanical properties of CSBR and CSBR/OMMT nanocomposite membranes.

Membrane	Tensile Strength (MPa)	Elongation at Break (%)	Modulus at 100% Elongation (MPa)
CSBR	3.45 ± 0.14	162.17 ± 13	1.75 ± 0.02
CSBR/OMMT3	3.54 ± 0.27	176.48 ± 03	2.08 ± 0.38
CSBR/OMMT6	3.63 ± 1.17	188.21 ± 27	2.37 ± 0.09
CSBR/OMMT9	4.02 ± 0.32	223.30 ± 17	2.47 ± 0.11
CSBR/OMMT12	4.89 ± 0.37	257.59 ± 09	2.71 ± 0.14
CSBR/OMMT15	6.25 ± 0.29	347.20 ± 26	3.00 ± 0.15

**Table 6:** The values of the crosslinking densities of pure SBR, CSBR and CSBR/OMMT nanocomposites.

Film Sample	Crosslink Density (mol·cm^−3^) × 10^−4^
pure SBR	0
CSBR	0.29
CSBR/OMMT3	1.04
CSBR/OMMT6	1.09
CSBR/OMMT9	0.99
CSBR/OMMT12	1.24
CSBR/OMMT15	3.22

**Table 7:** PV performance of CSBR/OMMT nanocomposite membranes.

Membrane	Total Flux (g m^−2 ^h^−^^1^)	Separation Factor	PSI (g m^−2 ^h^−^^1^)
CSBR/OMMT3	126.31	1.03	04.67
CSBR/OMMT6	182.56	1.08	14.78
CSBR/OMMT9	247.92	1.20	51.62
CSBR/OMMT12	260.67	1.43	113.81
CSBR/OMMT15	182.2	1.09	16.66

**Table 8:** Comparative results of the methanol/toluene mixture separation by pervaporation using different rubbery membranes taken from the literature.

Membrane	Toluene in the Feed (wt%)	T (°C)	J (g·m^−2^ h^−1^)	$\alpha_{PV}$	PSI (g·m^−2^ h^−1^)	Ref.
CSBR/OMMT12	32% (azeotropic composition)	30	260.67	1.43	113.81	This study
SBR-1 ^a^	0.55	30	12	162	1932	[34]
T5IL1 ^b^	27.2	24	75	128	9525	[39]
NR-3 ^c^	10.5	30	2.3	25	55.2	[34]
NR-20 ^d^	0.55	30	10	286	2850	[68]
NRZ10 ^e^	32	30	286.87	45.9	12,880.46	[33]
NRC10 ^f^	32	30	279.37	44.7	12,208.46	[33]
NRCB10 ^g^	32	30	256.87	41.1	10,300.48	[33]
NRZ	10.5	30	66.25	113.7	7466.37	[33]
NRC	10.5	30	73.75	105.9	7736.37	[33]
NRCB	10.5	30	78.12	102.7	7944.80	[33]
PDMS ^h^	32	25	95.36	2.67	159.25	[38]
(PU-PDMS) ^i^	32	25	113.14	3.66	300.95	[38]

^a^ SBR-1: SBR with conventional vulcanization; ^b^ T5IL1: ionic liquid modified MWCNT based SBR; ^c^ NR-3: efficiently vulcanized natural rubber; ^d^ NR-20: natural rubber containing 20 wt% N330 carbon black filler; ^e^ NRZ10: natural rubber containing 10 wt% zeolite; ^f^ NRC10: natural rubber containing 10 wt% cloisite filler; ^g^ NRCB10: natural rubber containing 10 wt% carbon black; ^h^ PDMS: polydimethyl siloxane rubber; ^i^ PU-PDMS: polydimethyl siloxane–polyurethane blend.

**Table 9:** Apparent diffusion coefficient of CSBR/OMMT nanocomposite membranes.

Membrane	*D_i_* × 10^−^^9^ m^2^·s^−1^	
	Methanol	Toluene
CSBR/OMMT3	0.11	0.30
CSBR/OMMT6	0.57	1.53
CSBR/OMMT9	1.30	3.58
CSBR/OMMT12	0.56	1.55
CSBR/OMMT15	0.81	1.93

## Data Availability

All the data supporting the findings of this study are available within the article.
